# The PTEN Conundrum: How to Target PTEN-Deficient Prostate Cancer

**DOI:** 10.3390/cells9112342

**Published:** 2020-10-22

**Authors:** Daniel J. Turnham, Nicholas Bullock, Manisha S. Dass, John N. Staffurth, Helen B. Pearson

**Affiliations:** 1 The European Cancer Stem Cell Research Institute, School of Biosciences, Cardiff University, Hadyn Ellis Building, Cardiff CF24 4HQ, UK; TurnhamD@cardiff.ac.uk (D.J.T.); bullocknp@cardiff.ac.uk (N.B.); DassMS@cardiff.ac.uk (M.S.D.); 2 Division of Cancer and Genetics, School of Medicine, Cardiff University, Heath Park, Cardiff CF14 4XN, UK; John.Staffurth@wales.nhs.uk

**Keywords:** PTEN, PI3K, targeted therapy, prostate cancer

## Abstract

Loss of the tumor suppressor phosphatase and tensin homologue deleted on chromosome 10 (PTEN), which negatively regulates the PI3K–AKT–mTOR pathway, is strongly linked to advanced prostate cancer progression and poor clinical outcome. Accordingly, several therapeutic approaches are currently being explored to combat PTEN-deficient tumors. These include classical inhibition of the PI3K–AKT–mTOR signaling network, as well as new approaches that restore PTEN function, or target PTEN regulation of chromosome stability, DNA damage repair and the tumor microenvironment. While targeting PTEN-deficient prostate cancer remains a clinical challenge, new advances in the field of precision medicine indicate that PTEN loss provides a valuable biomarker to stratify prostate cancer patients for treatments, which may improve overall outcome. Here, we discuss the clinical implications of PTEN loss in the management of prostate cancer and review recent therapeutic advances in targeting PTEN-deficient prostate cancer. Deepening our understanding of how PTEN loss contributes to prostate cancer growth and therapeutic resistance will inform the design of future clinical studies and precision-medicine strategies that will ultimately improve patient care.

## 1. Introduction

Prostate cancer is the second most frequently diagnosed male cancer worldwide, with over 1 million new cases recorded each year globally [1]. Although most men will not die from their disease, prostate cancer is a leading cause of male cancer-associated deaths, accounting for >350,000 deaths annually world-wide [1]. The primary cause of prostate cancer death is the emergence of incurable metastatic and castrate-resistant disease that is resistant to current treatment regimens, such as androgen-deprivation therapy (ADT) and chemotherapy, emphasizing the demand for new therapeutic strategies. In an emerging era of precision medicine, a comprehensive understanding of the molecular drivers of aggressive prostate cancer could improve patient outcomes through the tailored selection of targeted therapies, and identify patient populations with indolent, localized prostate cancer that are at risk of either developing aggressive forms of the disease or of being overtreated with potentially life-altering treatments.

Loss of the tumor suppressor PTEN is commonly observed in patients with aggressive, hard-to-treat prostate cancer. Although the frequency of PTEN loss in patients with prostate cancer varies between studies, largely owing to differences in the methodological approaches used, recent studies involving large prostate cancer patient cohorts have indicated that PTEN is lost in approximately 15–20% of primary prostate cancers [2,3,4,5]. A higher frequency of PTEN loss is observed in more aggressive stages of the disease including castration-resistant prostate cancers (CRPC) and metastatic disease, with PTEN lost in 40–60% of these cases [2,3,4,5,6]. The most common cause of functional PTEN loss in prostate cancer is genomic homozygous deletion of *PTEN*. However, several other mechanisms have also been reported, including inactivating somatic mutations (predominantly frameshift or non-sense truncating mutations), suppression of *PTEN*-targeting microRNAs (miRNAs) and inactivating post-translational modifications (e.g., phosphorylation and ubiquitylation) [7,8,9,10,11]. Infrequent epigenetic silencing via methylation of the *PTEN* promoter has also been observed in patients with prostate cancer [10,12,13].

Under normal physiological conditions, PTEN antagonizes phosphatidylinositol 3-kinase (PI3K) signaling through its role as a lipid phosphatase by converting phosphatidylinositol (3,4,5)-trisphosphate (PIP3), a lipid secondary messenger produced by PI3K, back to phosphatidylinositol (4,5)-bisphosphate (PIP2). Consequently, PTEN inhibits PIP3-mediated cellular events, including protein kinase B (PKB/AKT) and phosphoinositide-dependent kinase 1 (PDK1) signaling [14]. Functional loss of PTEN results in the accumulation of PIP3, which subsequently leads to unchecked PIP3 signaling that promotes oncogenic cellular events, including increased cell proliferation, survival and migration. To this end, PIP3 recruits PH domain-containing substrates to the membrane, such as AKT, which regulates a plethora of signaling components, including mammalian target of rapamycin (mTOR), glycogen synthase kinase 3 beta (GSK3β) and Forkhead box protein O (FOXOs) [14,15]. In addition to its role as a lipid phosphatase, PTEN can also exert its tumor-suppressive function as a protein phosphatase. Through direct protein interactions, primarily in the nucleus, PTEN is reported to directly regulate several cellular events including cell motility, chromosome stability, the DNA damage response and cell cycle dynamics [16,17,18,19].

PTEN loss of function has been extensively characterized as a driver of prostate tumor formation and progression using a range of experimental models [20,21,22,23,24,25]. Conditional biallelic deletion of *Pten* within the basal or luminal cell compartment of the mouse prostate causes invasive prostate carcinoma that recapitulates many features of the clinic, indicating that PTEN plays a tumor-suppressive role in the prostate epithelium [26]. Loss of PTEN has also been shown to cooperate with other genetic alterations including loss of the tumor suppressors (e.g., p53, RB, p27 or STAT3) and oncogenic mutations (e.g., *KRas*^G12D^, *BRaf*^V600E^ or *Pik3ca*^H1047R^) in vivo, resulting in accelerated disease progression, metastasis and/or de novo CRPC in response to castration/ADT [21,23,27,28,29,30,31,32]. Previous work in vivo has also shown that CRPC growth is an innate property of *Pten*-null prostate cancer cells, irrespective of tumor stage [33]. PTEN loss has also been linked to therapeutic resistance in the clinic, highlighting the need for further work to identify effective treatment options for patients with PTEN deficiency [34,35,36].

Given the high frequency of prostate cancers that exhibit PTEN loss with augmented PI3K–AKT–mTOR signaling, a significant amount of effort has been devoted to exploring PI3K–AKT–mTOR-directed therapies. Until recently, clinical trials investigating these agents have had limited success [37,38,39,40,41,42], reflecting several factors including dose-limiting toxicities and/or an inability to suppress the pathway. However, recent trials are beginning to show efficacy, owing to an improved molecular understanding of the complex regulatory framework within the PI3K–AKT–mTOR cascade, the development of new therapeutic agents/strategies and novel patient stratification approaches. Here, we review the present clinical landscape for patients with PTEN-deficient prostate cancer and summarize the current and promising therapeutic approaches being explored to treat this aggressive form of prostate cancer.

## 2. PTEN Status as a Predictive Biomarker for Prostate Cancer

The five-year survival rate for men with localized prostate cancer varies slightly depending on geographical location but has been reported to be as high as 98%, and some patients (1–10%) can outlive their disease without the need for invasive treatments [43,44]. Upon diagnosis, patients are stratified for treatments according to their risk classification. Conventional risk grouping for localized disease is based on serum PSA levels, tumor stage and Gleason score (GS), defined by the National Comprehensive Cancer Network (NCCN) risk groups (i.e., low risk: T1–T2a, GS ≤6 and PSA <10 ng mL^−1^; intermediate risk: T2b–T2c or GS 7 or PSA 10–20 ng mL^−1^; high risk: T3a or GS 8–10 or PSA >20 ng mL^−1^; high risk for locally advanced prostate cancer: T3b–T4 or primary Gleason pattern 5 or >5 cores with GS 8–10; and metastatic risk: N1/M1, with any T stage) [45], and recent refinements (using the International Society of Urological Pathology (ISUP) grading system and disease volume) are currently in clinical practice to aid treatment decisions [46]. Lately, there has been a sharp increase in the number of men with localized disease undergoing active surveillance [47]. However, active surveillance has recently been associated with significantly higher rates of tumor progression compared to those receiving proactive treatments [48]. These findings suggest that a subpopulation of active surveillance patients exists, which may benefit from more radical action. Importantly, genetic assessment of localized prostate cancer tissue biopsies is beginning to assist risk stratification and can simultaneously provide key molecular insight into the tumor biology to inform treatment decisions. Approved genomic tests for predicting risk in patients with localized disease include the Prolaris cell cycle progression score, the Decipher genomic classifier and the OncotypeDx genomic prostate score [49,50,51,52]. A string of studies have also established that PTEN status in localized prostate cancer serves as a valuable predictive marker for disease progression, which could improve patient selection for active surveillance and help guide treatment decisions [53,54,55,56,57]. For instance, immunohistochemistry (IHC) to detect PTEN in GS7 (3 + 4) biopsies has revealed that PTEN loss is associated with increased risk of non-organ confined disease at prostatectomy [54]. Furthermore, PTEN loss is strongly associated with advanced progression and poor outcome [34,35]. Together these findings indicate that low-risk patients with PTEN-deficient localized prostate cancer would benefit from radiotherapy or radical prostatectomy [2,58], although this treatment approach remains to be investigated in the clinic.

Several assays have been developed to assess PTEN status (+/− TMPRSS2:ERG fusion) to facilitate improved patient selection for active surveillance and/or treatment (e.g., Metamark, Cambridge, MA, USA) [5,59,60]. However, they are not currently used routinely in clinical practice. Common approaches to evaluate PTEN protein expression status include IHC and immunofluorescence (IF), while fluorescence in situ hybridization (FISH) and next-generation sequencing (NGS) assess *PTEN* genomic status. These methodologies have all independently identified that PTEN loss positively correlates with worse clinicopathological features and overall outcome for patients with prostate cancer [5,55,60,61,62]. Several studies have compared the accuracy of these tests, with IHC emerging as the predominant method of choice, as it is a straightforward, inexpensive and relatively robust assay [5,55,60]. Furthermore, analysis of PTEN protein expression by IHC has the added advantage of detecting PTEN loss that is not caused by a genomic alteration (such as miRNA and epigenetic silencing) and which is often missed by FISH analysis, especially in the context of PTEN loss of heterozygosity (LOH), which is present in 15–49% and up to 50% of localized and metastatic prostate cancers, respectively [23,63]. It has been suggested that FISH analysis should be performed when inconclusive IHC/IF results are observed. However, a dual testing strategy could also be employed whereby both IHC and FISH are implemented simultaneously, similarly to a new cost-effective HER2 screening strategy developed for invasive breast cancer [2,64]. Interestingly, new artificial intelligence-based algorithms are now being developed for automated detection and localization of PTEN loss, which have shown high accuracy in prostate cancer IHC stained sections and could further streamline PTEN screening [65]. Nevertheless, further work to better outline the criteria for assigning a tumor as PTEN-deficient is needed (e.g., PTEN cellular localization, PTEN conformational state, and the percentage of PTEN-negative prostate epithelial cells) [66,67,68,69]. In the literature, PTEN has been shown to localize to the cytoplasm and nucleus of normal basal and luminal prostate epithelial cells, and the surrounding stromal cells (including endothelial, smooth muscle, peripheral nerve, fibroblast and inflammatory cells) [5,6,26,70,71], while others have additionally observed PTEN at the membrane [5,72]. These variations are likely to reflect differences in PTEN function, but could also relate to differences in the methodologies, such as the tissue fixation protocol and/or the antibody binding site.

**Figure 1:**
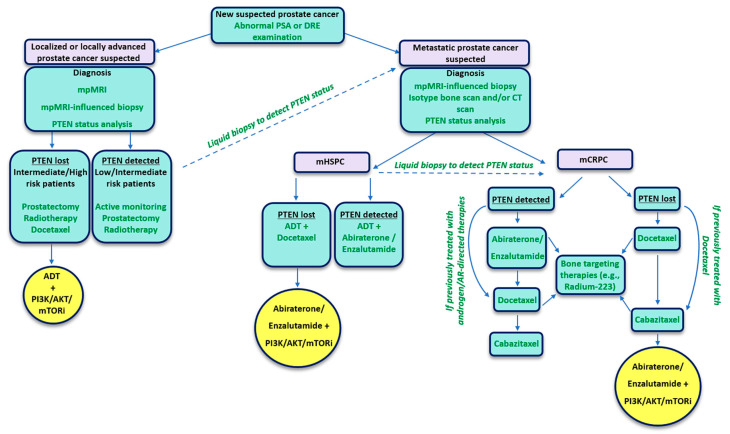
Tailoring treatments for local and metastatic prostate cancer based on PTEN status. Image shows hypothetical guidelines for diagnosing and treating prostate cancer following PTEN screening. Following an abnormal PSA and/or digital rectal examination (DRE), men with suspected prostate cancer would be advised by a specialist regarding the potential risks and benefits of the various treatment options before being diagnosed following current NICE guidelines. For suspected localized or locally advanced prostate cancer, multiparametric magnetic resonance imaging (mpMRI) scans should be used where available as first-line investigation, with patients scoring 3 or more on the Likert scale offered a mpMRI-influenced prostate biopsy. Alongside normal assessment of PSA, the Gleason score and clinical stage, additional analysis of PTEN status by IHC and/or FISH should help determine appropriate risk category by multidisciplinary teams. Those with low- or intermediate-risk localized disease with normal PTEN expression can be offered a choice of active surveillance, radical prostatectomy or radiotherapy. Patients with intermediate localized or high locally advanced disease with PTEN loss should not be offered active surveillance and may be treated with docetaxel if high risk with no significant comorbidities. ADT therapy plus PI3K–AKT–mTOR-targeted therapies may be beneficial for high-risk patients where PTEN has been lost but this has not been tested clinically. If metastatic disease is detected in patients who have progressed from locally advanced disease, PTEN status should be re-assessed by analysis of CTCs if previous biopsy analysis detected normal PTEN status. Newly diagnosed metastatic disease or for those who have not received a mpMRI biopsy should also have CTCs assessed to determine PTEN status. Patients with mHSPC and normal PTEN expression should proceed with ADT alongside second-generation androgen/Androgen Receptor (AR)-targeting therapies such as abiraterone or enzalutamide, but if PTEN has been lost, docetaxel should be used as a first-line treatment alongside ADT as PTEN loss has been shown to promote resistance to next-generation hormonal therapies. The use of AR-targeted therapies may be efficacious alongside PI3K–AKT–mTOR inhibition, although this needs to be extensively tested clinically. As mHSPC progresses to mCRPC, patients should be retested for loss of PTEN expression in CTCs if they were previously diagnosed with PTEN-positive disease and then proceed to taxane-based treatments if they have previously received abiraterone and/or enzalutamide and are still presenting PTEN-positive disease. For newly presenting mCRPC where PTEN is lost, taxane-based therapies should also be used upfront before experimental PI3K–AKT–mTOR inhibitors are used alongside abiraterone or enzalutamide. ADT, androgen-deprivation therapy; CT scan, computerized tomography scan; DRE, digital rectal exam; mpMRI, multiparametric magnetic resonance imaging; mCRPC, metastatic castrate-resistant prostate cancer; mHSPC, metastatic hormone-sensitive prostate cancer; PI3K/AKT/mTORi, PI3K/AKT/mTOR inhibitor; PSA, prostate-specific antigen.

Investigations to identify a PTEN-deficient gene signature are now underway, which could overcome some of the limitations associated with the methodologies outlined above [73]. However, the issue of tissue heterogeneity remains, as analysis of needle biopsies does not always represent the whole tumor. Interestingly, liquid biopsy analysis of *PTEN* genomic status within circulating tumor cells (CTCs) is also being investigated as a quick, non-invasive strategy to identify patients with PTEN-deficient prostate cancer, with a view to inform treatment decisions [74,75]. For instance, a blood-based CTC PTEN FISH assay is being employed in a clinical trial exploring abiraterone in combination with PI3K/AKT inhibitors in patients with CRPC (NCT01485861) [74]. Importantly, PTEN loss in CTCs isolated from patients with mCRPC has been shown to significantly correlate with reduced survival in two independent studies [74,75], and several studies have revealed that PTEN loss can influence response to current therapies [34,35,76,77,78,79]. Indeed, a recent large phase II trial in breast cancer is already using CTC DNA analysis to match key driver mutations in CTCs, including PTEN inactivating mutations, to select personalized therapies for patients, such as AKT-targeted therapy for PTEN-deficient patients [80]. Thus, the routine analysis of PTEN status may not only identify patients at risk of developing more aggressive disease, but could also be used to select more personalized treatments (Figure 1).

High levels of intertumor and intratumor heterogeneity are a hallmark of prostate cancer, which makes the selection of an appropriate and efficacious therapeutic approach difficult [81]. Notably, *PTEN* deletion is frequently observed in primary prostate tumors with high levels of intratumor heterogeneity [82], and the heterogeneity of PTEN loss within a primary prostate tumor has also been shown to influence clinical outcome [83]. Lotan and colleagues assessed PTEN status by IHC in a large radical prostatectomy tissue microarray, revealing that relative to cores with intact PTEN, PTEN homogeneous loss, but not heterogeneous loss, is significantly associated with shorter recurrence-free survival (RFS) in multivariate models [83]. At the genomic level, both heterozygous and homozygous deletion of *PTEN* significantly associate with biochemical recurrence (based on rising PSA levels) and RFS in patients that have undergone radical prostatectomy or brachytherapy [76]. *PTEN* copy number loss has also been associated with disease progression following radiotherapy [77], which has been linked to the increased infiltration of tumor-associated macrophages (TAMs) in PTEN-deficient tumors, which release pro-survival signals that could provide beneficial therapeutic targets [84]. It has also been observed that in localized high risk disease, patients with PTEN loss relapse quicker when treated with chemotherapy post-surgery compared to those with intact PTEN [78]. However, PTEN status has been shown to have little effect on taxane-based chemotherapy response in patients with mCRPC [36]. Finally, treatment pressure can also lead to the acquisition of *PTEN* loss, as observed in *PIK3CA* mutant metastatic breast cancer patients that developed resistance to the p110α-specific PI3K inhibitor apelisib (BYL719) [85]. Here, *PTEN* loss is thought to reactivate the PI3K cascade via p110β to overcome p110α inhibition. Taken together, these findings further highlight the predictive value of PTEN status in prostate cancer and emphasize the need for future work to identify beneficial stratification strategies for PTEN-deficient prostate cancer in order to better inform clinical trial design and improve patient care. To this end, improving our molecular understanding of the functional consequence of PTEN loss during prostate cancer growth and in response to therapeutic intervention is paramount.

The standard of care for patients that present with mHSPC is shifting towards earlier treatment of ADT in combination with abiraterone, enzalutamide or docetaxel, with several landmark clinical studies highlighting the significant clinical benefits of these combined therapeutic strategies compared to ADT alone [86,87,88,89,90,91]. Despite these promising results, the challenge remains to further stratify patients towards drug combinations that are likely to offer the most benefit. PTEN loss could become a useful biomarker in this setting, as patients whose prostate cancer is maintained through ADT are more likely to progress quickly to lethal mCRPC if PTEN has been lost [35,92,93]. Furthermore, loss of PTEN has been shown to downregulate AR activity and can promote resistance to next-generation hormonal therapies such as abiraterone. Therefore, PTEN-deficient patients with advanced prostate cancer may benefit more from taxane-based therapies [33,34,36]. In the mHSPC setting upfront ADT alongside docetaxel could be the best approach to offer long-lasting responses following PTEN loss, while similarly in mCRPC, taxane-based therapies are likely to be the most effective upfront therapy compared to second-generation AR-targeting treatments (Figure 1). New data are now beginning to suggest that second-generation hormonal therapies may be more effective in PTEN-deficient mCRPC when used alongside PI3K–AKT–mTOR inhibition (discussed below). However, these approaches will require further clinical evaluation before being introduced as a standard treatment option. Whether the success of these new combinations may also translate into the mHSPC setting remains to be established. However, a new clinical trial to explore this is now actively recruiting (NCT04493853).

## 3. Targeting PTEN-Deficient Prostate Cancer

Given the high frequency of PTEN loss in prostate cancer, therapeutic strategies that exploit the functional loss of PTEN may prove to be efficacious against PTEN-deficient prostate cancer. Below, we discuss the recent advances in the development of agents that can directly restore PTEN function and the therapeutic approaches to inhibit the PI3K–AKT–mTOR signaling network.

### 3.1. Direct Restoration of PTEN Function

**Figure 2:**
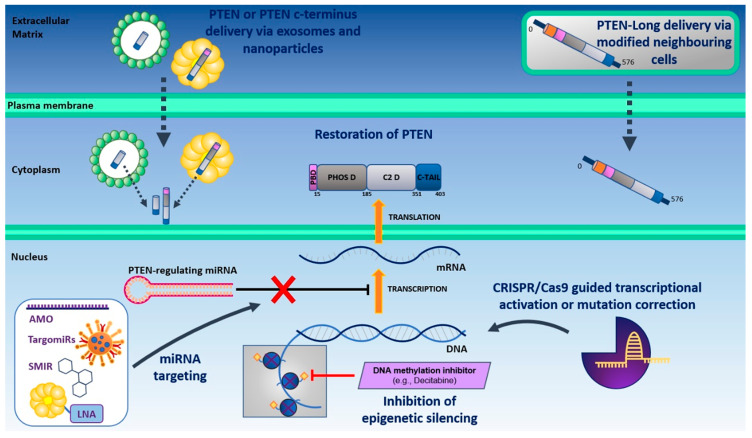
Experimental therapeutic approaches to restore PTEN functional dose. AMO, anti-miR oligonucleotide; C2 D, C2 domain; LNA, locked nucleic acid; Cas9, CRISPR associated protein 9; CRISPR, clustered regularly interspaced short palindromic repeat; miRNA, micro ribonucleic acid; PBD, PIP2-binding domain; Phos D, phosphatase domain; PTEN, phosphatase and tensin homologue deleted on chromosome 10; SMIR, small-molecule inhibitors of miRNA.

In theory, perhaps the simplest approach to overcome PTEN-deficient prostate cancer is to restore PTEN function. Multiple lines of evidence in the literature have indicated that PTEN plays a haploinsufficient tumor-suppressive role. Transgenic mouse models have shown that *PTEN* haploinsufficiency or heterozygosity in mouse prostate epithelium can cause prostate lesions, whereas homozygous *PTEN* deletion causes prostate carcinoma [20,23,94]. Moreover, PTEN loss has been shown to cooperate with other oncogenic events to accelerate prostate cancer progression in vivo in a dose-dependent manner, and heterozygous loss of PTEN has been shown to accelerate prostate cancer progression in some clinical datasets [22,76,79,95,96,97]. These findings underpin the notion that artificially increasing the ‘functional dose’ of PTEN, even slightly, could elicit a significant reduction tumor burden and/or the rate of caner progression by restoring PTEN tumor-suppressive functions, thus presenting an attractive therapeutic approach. Accordingly, several novel treatment strategies are being developed to directly restore PTEN function (Figure 2), as outlined below.

#### 3.1.1. Direct Delivery of PTEN and PTEN-Long

To increase the functional dose of PTEN in PTEN-deficient prostate cancer, experimental therapeutic approaches designed to deliver PTEN directly into prostate cancer cells are currently being studied. Historically, the systemic delivery of macromolecules such as PTEN has been challenging, largely due to issues such as high renal clearance, rapid protein degradation and poor membrane permeability limiting intracellular uptake [98]. However, the recent, rapid advancement of protein modification techniques and novel delivery systems (e.g., micro and nanoparticle encapsulation) has made the delivery of PTEN into tumor cells feasible [98]. Significantly, intracellular nanoparticle assisted delivery of recombinant PTEN protein has been successfully used in PTEN-deficient prostate cancer cell lines in vitro to reduce cell viability, and PTEN has been shown to accumulate at PC-3 xenografts in vivo using a nanoparticle delivery system [99,100]. A similar approach has also been used to deliver *PTEN* mRNA, yielding promising results in PC-3 tumor models in vivo, reducing both primary and metastatic tumor growth through suppression of the PI3K–AKT signaling pathway [101]. These findings suggest that this therapeutic approach may also prove to be efficacious in patients. Interestingly, in vitro studies have shown that PTEN can also be secreted via exosomes, which can then be taken up by surrounding recipient cells to reduce AKT activity and cell proliferation [102]. This system could be exploited therapeutically, and encouragingly exosome-based delivery of the C-terminus region of PTEN (that stabilizes PTEN) has shown anti-tumor effects in breast cancer models, both in vitro and in vivo [103]. Consequently, this approach could transpire to be particularly beneficial in patients with *PTEN* mutations in this hotspot region.

The discovery of PTEN-Long has added an additional therapeutic opportunity to reinstate the tumor-suppressive function of PTEN to treat PTEN-deficient cancers. PTEN-Long is a translational variant of PTEN that includes an additional 173 amino acids at its N-terminus (N-173), which allows it to be secreted and taken up by surrounding cells to suppress PI3K–AKT–mTOR signaling and induce tumor cell death [104]. Although little work has been performed to harness the therapeutic potential of PTEN-Long in prostate cancer, purified PTEN-Long (but not purified PTEN) has been shown to inhibit tumor proliferation and induce tumor regression in a clear cell renal cell carcinoma xenograft model in vivo [105]. Furthermore, an altered version of PTEN-Long engineered to improve secretion efficiency has been investigated in a cell-mediated protein delivery system, which showed biological activity in vitro against neighboring glioblastoma cell lines [106]. Taken together, these preclinical studies highlight the therapeutic potential of PTEN-Long delivery to treat PTEN-deficient epithelial cancers. However, further work is needed to gain insight into the most beneficial approach for PTEN-deficient prostate cancer (e.g., PTEN vs. PTEN-Long delivery). To this end, increased molecular characterization of the mode of action of PTEN-Long is needed to determine tissue specificity and to establish the existence/functional importance of the PTEN-independent functions of PTEN-Long, especially given that the PTEN-Long N-173 tail is considered to harbor multiple protein-binding sites [107].

#### 3.1.2. Restoring PTEN Function by Targeting PTEN-Negative Regulators

Restoration of PTEN expression by overcoming its transcriptional and post-transcriptional repression is also being investigated as an avenue for therapeutic intervention to increase PTEN activity (Figure 2). Although PTEN is frequently lost through genomic alterations, post-transcriptional acetylation, ubiquitination and phosphorylation of PTEN have also been shown to impair PTEN stabilization, localization and/or phosphatase activity, while a number of regulatory proteins have also been shown to interact with PTEN at various binding sites to mediate PTEN activity (reviewed in [108]). For example, the cytosolic mannosidase α-mannosidase 2C1 (MAN2C1) has been shown to interact with and negatively regulate the lipid phosphatase activity of endogenous PTEN, and MAN2C1 overexpression stimulates AKT signaling and promotes prostate tumorigenesis in PTEN-positive DU145 prostate cancer xenografts [109]. Furthermore, PTEN-positive prostate cancers frequently overexpress MAN2C1, and MAN2C1 expression in prostate cancer significantly correlates with reduced recurrence-free survival [109]. Although limited research has explored the therapeutic benefit of targeting MAN2C1, silencing of MAN2C1 by siRNA has been shown to induce apoptosis in esophageal carcinoma cells [110], while its suppression in nasopharyngeal carcinoma cells has been demonstrated to inhibit tumor formation and metastasis in vivo [111]. These findings illustrate the therapeutic benefit of targeting proteins that negatively regulate PTEN post-translation, and corroborate the need for future work to comprehensively investigate the therapeutic benefit of targeting PTEN-negative regulators in prostate cancer with inactive PTEN, and to establish their true predictive value.

#### 3.1.3. miRNA Targeting to Restore PTEN Transcriptional Activity

*PTEN* expression is also regulated post-transcriptionally by non-coding oncogenic microRNAs (miRNAs) known as oncomiRs. To date, oncomiRs such as miR-17, miR-20a, miR-21, miR-22, miR-25, miR-93, miR-106a/b, miR-153, miR-498 and miR-4534 have each demonstrated the ability to downregulate *PTEN* expression in prostate cancer cells, thus presenting potential therapeutic targets [112,113,114,115,116,117,118,119,120]. Perhaps the most promising preclinical candidate for restoring PTEN function is miR-21, which has been successfully silenced using antisense anti-miR oligonucleotides (AMOs) and locked nucleic acid (LNA) conjugated to lipid nanocapsules in other cancer types [121,122]. Additional methods to inhibit oncomiRs include small-molecule inhibitors of miRNAs (SMIRs), anti-miRs, antagomiRs and bacterial-based TargomiRs (Figure 2) [123,124]. With clinical trials investigating LNA anti-miRs, TargomiRs and liposomal packaged miRNAs designed to target various oncomiRs in solid tumors beginning to recruit, and initial reports showing relatively good safety profiles, this approach could be readily applied to multiple cancer types with PTEN deficiency in the near future, including prostate cancer [123,125,126,127]. Nevertheless, the mechanism through which PTEN has been lost and off-target effects remain two important considerations for future clinical trials investigating the targeting of oncomiRs to treat PTEN-deficient prostate cancers [128].

Lastly, although epigenetic silencing of *PTEN* in prostate cancer is uncommon, demethylating agents able to restore *PTEN* expression may also offer a beneficial therapeutic approach for patients with *PTEN* silencing [129]. For instance, in a non-small-cell lung carcinoma cell line the demethylating agent decitabine (5-aza-2′-deoxycytidine) was able to increase *PTEN* mRNA levels, while knockdown of the DNA methyltransferase enzyme DNA (cytosine-5)-methyltransferase 3A *(DNMT3A*) in a hepatocellular carcinoma cell line resulted in demethylation of the *PTEN* promoter and upregulation of *PTEN* mRNA expression [130,131]. Although there is a lack of evidence for the direct restoration of PTEN through manufactured demethylating agents in *PTEN*-silenced prostate cancer, the naturally occurring dietary compound resveratrol has been shown to promote PTEN acetylation and reactivation by inhibiting an interaction between the metastasis-associated protein 1 (MTA1) and histone deacetylase (HDAC) complex in prostate cancer cells [132].

#### 3.1.4. CRISPR/Cas9-Guided Transcriptional Activation of PTEN

The use of genome editing technology to directly reactivate *PTEN* is a relatively new approach being investigated to treat PTEN-deficient cancer that is not caused by a genetic alteration in *PTEN*, although this approach may only be applicable for up to 10% of PTEN-deficient prostate cancers in which *PTEN* transcription is suppressed by negative transcriptional regulators of PTEN or via promoter methylation [2]. Recent work has shown that CRISPR/dCas9 gene editing can be been used to activate *PTEN* transcription in breast cancer and melanoma cell lines that harbor wild-type *PTEN* alleles but have low *PTEN* expression in vitro [133]. Here, a kinase dead Cas9 (dCas9) fused to a VPR (VP64, p65, and Rta transactivator) effector domain (termed dCas9-VPR activation system) was used to target the proximal promoter region of *PTEN*, resulting in increased *PTEN* mRNA levels and PTEN protein expression, which suppressed downstream AKT/mTOR signaling [133]. Importantly, no mRNA expression changes were observed in previously identified potential off-target genes in this setting. However, future work focusing on the delivery of these constructs in vivo will be vital for advancing their clinical utility. In addition, CRISPR/Cas9 technology has also been employed to alter the start codon of *PTEN-Long* in HEK293 cells, resulting in a significant increase in PTEN-Long expression and secretion [134]. Remarkably, the transfer of conditioned media from these cells to cultured PTEN-null glioblastoma U87 cells resulted in the uptake of PTEN-Long, which led to reduced AKT signaling and cell proliferation [134]. Although transcriptional activation of *PTEN* is yet to be achieved in vivo, CRISPR/Cas9-mediated repression of *PTEN* in mouse models has demonstrated the ability of this system to target PTEN in vivo to rapidly induce *PTEN*-deficient murine liver disease and mammary tumors [135,136,137]. Future studies to determine whether CRISPR/Cas9-guided therapeutic approaches in patients with low *PTEN* expression that carry a monoallelic genetic aberration in *PTEN* will be of great interest to the field, possibly extending the application of this therapeutic strategy to a larger population of patients.

Notably, Cas9-nickases that induce DNA single-strand breaks offer a potential tool to correct somatic *PTEN* mutations, as they can be modified to introduce single nucleotide base changes, and this approach has previously been shown to correct *TP53* mutations in breast cancer cell lines [138]. Genome editing has been used in a number of clinical trials for various pathological conditions. However, the majority of these genetic modifications have been undertaken ex vivo using patient or donor cells, which are then engrafted into the patient [139]. To target *PTEN* genetic alterations in patients with prostate cancer, an in vivo approach is needed. However, several non-trivial hurdles will need to be overcome first, including the efficiency of delivery, immunogenicity and off-target effects [139,140]. To date, in vivo approaches have focused on readily accessible tissues, with direct targeting of the prostate likely to be a significant challenge. Both viral and aptamer-liposomal systems have been used to deliver CRISPR guides specifically to the prostate epithelium in mice, with the latter showing promising anti-tumor efficacy and a limited immune response, suggesting that this could be a viable option in the future [141,142].

In conclusion, a wide range of therapeutic approaches to restore PTEN activity are in development, which target multiple PTEN-negative regulation events. As this field continues to rapidly grow and delivery mechanisms significantly improve, these types of approaches could become much more feasible, opening the door to an exciting and potentially highly effective method of restoring functional PTEN in PTEN-deficient prostate cancer. Advantageously, this approach could also overcome the complex mechanisms of resistance that have hindered other therapeutic strategies targeting the PI3K–AKT–mTOR cascade (discussed below). However, the co-occurrence of other genetic drivers of the PI3K pathway with PTEN loss, and the cause of PTEN loss would need to be carefully considered when selecting patients for treatment. Further work to establish an in-depth understanding of the molecular mechanism by which PTEN is regulated would provide important insights into how PTEN-negative regulation can be most effectively targeted, and studies to identify which patients are most likely to benefit from restoring PTEN activity by targeting specific PTEN regulation events are warranted.

### 3.2. PI3K Inhibition

Oncogenic PI3K signaling frequently occurs in prostate cancer, and is invariably activated in metastatic disease [21,143,144,145]. The PI3Ks are a family of intracellular signal transduction enzymes that can be separated into 3 structurally and functionally defined classes (Class I, that is subdivided into Class 1A and Class 1B, Class II and Class III). Here, we focus on Class1A PI3Ks, which have been shown to contribute to prostate tumor formation and progression [146,147] (for more information on Class II and Class III PI3Ks refer to [144,148]). Class IA PI3Ks function as heterodimers comprised of a catalytic isoform (p110α, p110β, and p110δ) and a regulatory subunit (p85α, p85β, p55α, p55γ or p50α) that catalyze the convsion of PIP2 to PIP3 upon recruitment to the cytoplasmic domain following the activation of upstream receptors (e.g., receptor tyrosine kinases, RTKs, G-protein coupled receptors, GPCRs) and small GTPases [147]. PTEN is a master regulator of the PI3K cascade, that antagonizes PI3K catalytic activity and therefore restricts PIP3-mediated signaling [149]. PIP3 recruits several effector proteins that bind via a pleckstrin homology domain (PH domain), including AKT and phosphoinositide-dependent kinase-1 (PDK1). Upon recruitment, AKT is subsequently phosphorylated at Thr308 by PDK1, triggering a wave of AKT-mediated phosphorylation events that mediate cell survival, proliferation, glucose metabolism and ribosomal biogenesis through multiple downstream effector pathways (e.g., mTORC1 signaling) [150]. Further phosphorylation of AKT at Ser473 by mTORC2 results in AKT hyperactivation [150,151].

**Figure 3:**
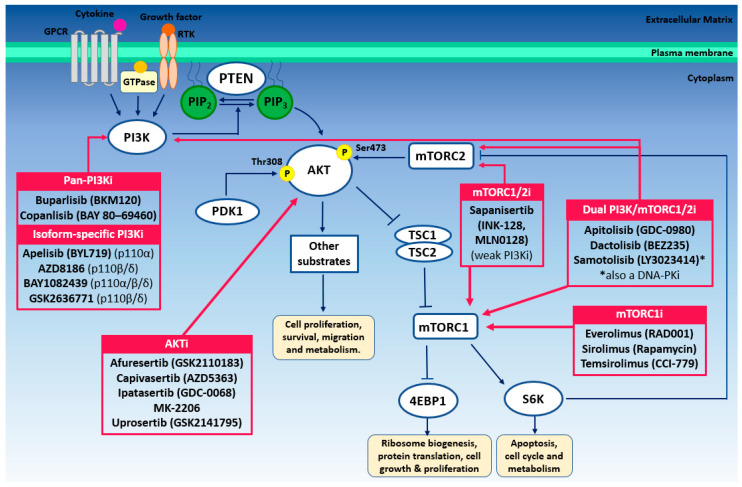
Targeting the PI3K–AKT–mTOR pathway to treat prostate cancer. Image shows small-molecule inhibitors that target key components of the PI3K–AKT–mTOR signaling pathway that have recently been investigated in the clinic to treat prostate cancer. 4EBP1, Eukaryotic translation initiation factor 4E-binding protein 1; AKTi, AKT inhibitor; DNA-PKi, DNA-dependent protein kinase inhibitor; GPCR, G-protein coupled receptor; mTORC1/2, mammalian target of rapamycin complex 1/2; mTORC1i, mTORC1 inhibitor, mTORC1/2i, mTORC1/2 inhibitor, P, phosphorylation event; Pan-PI3Ki, pan-PI3K inhibitors; PDK1, phosphoinositide-dependent kinase 1; PI3K, phosphoinositide 3-kinase; PIP2, phosphatidylinositol 4,5-bisphosphate; PIP3, phosphatidylinositol 3,4,5-trisphosphate; RTK, receptor tyrosine kinase; S6K, p70 ribosomal S6 kinase; TSC1, tuberous sclerosis complex 1; TCS2, tuberous sclerosis complex 2.

In prostate cancer, activation of the PI3K–AKT–mTOR cascade occurs through multiple mechanisms (reviewed in [144]). Although PTEN loss is the most frequent cause, a range of distinct genetic alterations can also cause oncogenic signaling of this pathway. These include activating mutations in *AKT* and *PIK3CA/PIK3CB*, which encode the p110α/p110β catalytic PI3K subunits, respectively [152,153]. Interestingly, genetic alterations in *PIK3CA* have a strong tendency to coexist with *PTEN* deep deletion/mutation in patients with prostate cancer, and we have previously shown in mice that *Pten* homozygous deletion and an activating mutation in *Pik3ca* at H1047R synergize to promote prostate cancer progression and de novo CRPC [21]. The high frequency of deregulated PI3K–AKT–mTOR signaling has led to the development of several small-molecule inhibitors designed to target individual p110 PI3K catalytic isoforms as well as pan-PI3K inhibitors, AKT inhibitors, mTOR inhibitors, and a number of dual action PI3K/mTOR inhibitors (Figure 3) [154].

Although early clinical trials involving these inhibitors were terminated due to substantial toxicity issues and a lack of on-target efficacy, several recent trials are beginning to show promising results in a variety of cancer types, including prostate cancer [154]. Initial studies with the non-specific PI3K antagonist LY294002 suggested that PI3K inhibition could inhibit invasion [155], angiogenesis [156] and sensitize prostate cancer cells to radiation therapy [157]. However, this agent has not entered clinical evaluation due to severe toxicity observed in mice [158]. The pan-PI3K inhibitor buparlisib (BKM120) is, however, well tolerated in mice, and in vivo preclinical studies in a variety of cancer types have reported anti-tumor efficacy associated with reduced AKT activity in response to buparlisib treatment, including prostate cancer models [21,159,160,161,162]. A phase 1 study in men with high-risk, localized prostate cancer using buparlisib pre-prostatectomy confirmed that buparlisib can inhibit AKT activity (NCT01695473), yet no change in cell death or proliferation was observed [163]. The pan-PI3K inhibitors copanlisib (BAY 80–69460) and pilaralisib (XL-147) have also been shown to decrease PI3K activity in vitro, with the latter showing a significantly higher potency in PTEN-deficient prostate cancer cell lines [164,165]. Interestingly, one clinical study is currently recruiting to test the efficacy of copanlisib in combination with the PARP inhibitor rucaparib in mCRPC (NCT04253262). However, to our knowledge, pilaralisib is yet to be investigated clinically to treat prostate cancer. Dactolisib (BEZ235), a dual PI3K/mTORC1/2 inhibitor has also shown good anti-tumor activity in various preclinical models, including *PTEN*-null prostate cell lines [159,166]. However, clinical testing in several cancer types, including one study in mCRPC in combination with abiraterone (NCT01717898), has been terminated early owing to dose-limiting toxicities [38,167].

A range of highly potent PI3K catalytic isoform-specific inhibitors have also been developed, providing an opportunity to reduce the therapeutic dose administered and limit toxicity [154]. While *PIK3CA* mutant prostate cancers have been shown to respond to p110α isoform-specific inhibition, previous work by us and others has shown that targeting p110α alone in prostate cancers with PTEN loss is not efficacious, owing to p110β dependency [21,144,168,169]. Although acquisition of *PTEN* genetic alterations in response to the p110α inhibitor alpelisib (BYL719) have been shown to drive resistance in metastatic breast cancer patients [85], indicating that similar mechanisms of resistance may emerge in PTEN-deficient prostate cancer, p110α inhibition may still prove to be efficacious against prostate cancers that are both PTEN-deficient and carrying an activating *PIK3CA* mutation. Whether combining p110α- and p110β-specific inhibitors is more efficacious and/or less toxic than a pan-PI3K inhibitor is also yet to be explored clinically.

The reliance of PTEN-deficient prostate cancers on p110β activity underpins the rationale for clinical trials exploring the efficacy of p110β-targeted inhibition. The p110β-selective inhibitor GSK2636771 (which also selectively targets p110δ, albeit less potent) has shown promise in mCRPC in a first-in-human trial (NCT01458067) [170]. In addition, GSK2636771 combined with enzalutamide in PTEN-deficient mCRPC patients is reported to be well tolerated, with 1/13 evaluable patients showing a partial radiological response and, encouragingly, another two patients displayed maximum PSA reductions (>50%) (NCT02215096) [171]. Co-targeting p110β and p110δ in mCRPC is also being explored using the dual p110β/p110δ PI3K inhibitor AZD8186 +/− abiraterone (with prednisone), plus an expansion phase with *PTEN*-deficient/mutated or *PIK3CB*-mutated mCRPC (NCT01884285). A phase I trial is also studying the efficacy of AZD8186 in combination with docetaxel in *PTEN* or *PIK3CB* mutant mCRPC patients (NCT03218826). Preclinically, two additional p110β-specific inhibitors KIN-193 (AZD6482) and SAR260301 have also shown efficacy in various murine *PTEN*-deficient cancer models in vivo [172,173], further supporting the therapeutic strategy of targeting p110β in cancers with PTEN loss. Nevertheless, clinical evaluation has not progressed beyond phase 1 for either drug. SAR260301 was unable reduce AKT signaling due to rapid clearance (determined by an ex vivo platelet p-AKT-inhibition assay) (NCT01673737) [174], and AZD6482 has not been followed up clinically in an anti-tumor setting since its initial phase 1 safety and tolerability study performed >10 years ago (NCT00688714). The p110β isoform-specific PI3K inhibitor TGX-221 and its analogue BL140 have also both shown efficacy in preclinical models of prostate cancer with *PTEN* loss [175,176,177,178,179], and BL140 is also reported to overcome resistance to enzalutamide in vitro [179]. Controversially, however, previous work by us indicates that TGX-221 treatment alone in a *Pten*-deficient prostate cancer transgenic mouse model is not sufficient to significantly reduce tumor burden, although tumor regression was observed when TGX-221 was combined with the p110α isoform-specific PI3K inhibitor A66 [21], indicating that p110β inhibition may shift dependency to p110α in this setting [21,168]. Consequently, further work is needed to improve our understanding of the compensatory mechanisms between p110 isoforms in response to p110 isoform-specific blockade to improve future clinical trial design, and these findings provide additional support for future studies exploring p110α and p110β isoform-specific inhibitor combination therapy in patients with PTEN loss.

Several mechanisms of resistance to PI3K inhibitors have been identified, including hormone receptor dependency, activation of downstream kinases, gene expression deregulation via aberrant transcription factor signaling and increased reliance on alternative p110 isoforms which can each help drive cell growth [180]. To overcome PI3K inhibitor resistance, a number of combination therapy approaches have been explored. Previous work has revealed that the dual PI3K/mTOR inhibitor dactolisib (BEZ235) promotes AR signaling in a HER2/3 dependent manner in *PTEN*-deficient prostate cancer models, while AR-targeted therapy augments AKT signaling [181]. This important study identified a reciprocal feedback loop exists between the PI3K and AR signaling pathways, and that relative to monotherapy, combined dactolisib and enzalutamide (MDV3100) treatment shows superior efficacy in both a *Pten*-deficient murine prostate cancer model and human prostate cancer xenografts [181]. These data provide a clear justification for combining PI3K pathway (or HER2 kinase) inhibition with anti-androgen/AR-directed therapy in the clinic, and multiple clinical trials are now exploring PI3K and AR pathway co-inhibition in mCRPC [37,182,183]. However, a phase 1b study testing dactolisib or buparlisib in combination with abiraterone was terminated early due to dose-limiting toxicity including hyperglycemia, a lack clinical efficacy and a poor pharmacokinetic profile [183], and a phase 2 study combining buparlisib and enzalutamide in mCRPC patients saw no significant PSA reductions and no radiographic responses in the 30 men tested [37]. Nonetheless, combining the dual PI3K/mTOR and DNA-dependent protein kinase (DNA-PK) inhibitor Samotolisib (LY3023414) with enzalutamide to treat mCRPC that has progressed on abiraterone has shown early promise, and a manageable safety profile (NCT02407054) [182]. The median radiographic progression-free survival (rPFS) was 7.5 months compared to 5.3 in those treated with enzalutamide alone (HR ratio = 0.68), and patients with androgen receptor variant 7 (AR-V7)-negative disease showed a remarkable increased in rPFS (13.2 months, HR ratio = 0.52) [145]. Upcoming results from a trial exploring the dual PI3K/mTOR kinase inhibitor apitolisib (GDC-0980) in combination with abiraterone (NCT01485861) are also expected to provide further insight into the therapeutic benefit of this co-targeted approach. The efficacy of the dual PI3K/HDAC inhibitor fimepinostat (CUDC-907) is also currently being assessed clinically in various solid tumor types and although this does not currently include patients with prostate cancer, fimepinostat is reported to reduce tumor growth in the *PTEN*-deficient LuCaP 35CR PDX CRPC model [184,185].

While p110δ signaling has been predominantly shown to play an oncogenic role in hematological malignancies [148], mRNA levels of *PIK3CD* (that encodes p110δ) are reported to increase in prostate carcinoma compared with normal prostate, and PI3K pathway activation in *PTEN*-deficient prostate cancer cells has been shown to involve both p110β and p110δ PI3K signaling [186,187]. These data suggest that targeting the p110δ catalytic PI3K isoform may be efficacious in prostate cancer in combination with the inhibition of other p110 catalytic PI3K isoforms. In addition to the p110β/δ-selective inhibitors discussed above, the selective p110α/β/δ PI3K inhibitor BAY1082439 is reported to inhibit tumor growth in a bi-allelic *Pten*-deficient prostate cancer transgenic mouse model, and can prevent invasive progression and reduce epithelial-to-mesenchymal transition (EMT) in the *Pten*-deficient, *KRas^+/G12D^* mutant mouse model of metastatic prostate cancer [188]. Notably, BAY1082439 was shown to target both prostate tumor cells intrinsically and the surrounding tumor microenvironment, as targeting p110δ also blocks B-cell infiltration and lymphotoxin release in the tumor microenvironment that can promote CRPC growth [188]. A phase 1 dose-escalation study to assess the safety and tolerability of BAY1082439 in patients with advanced solid malignancies has now been completed (NCT01728311). However, no results from this study have been disclosed to date.

### 3.3. AKT Inhibition

In the absence of PTEN, PI3K activity results in the uncontrolled production of the phospholipid second messenger PIP3, resulting in the activation of several downstream effector proteins such as the serum/glucocorticoid-regulated kinases (SGKs), protein kinase C (PKC), Rho family GTPases and the well-characterized AKT signaling cascades to mediate cell growth, proliferation and migration [189,190,191]. AKT is a serine/threonine protein kinase, of which three structurally similar isoforms exist (AKT1, AKT2 and AKT3) that are differentially expressed across tissue compartments, with AKT1 being the most abundantly expressed across most tissues [192]. Activating mutations in *AKT1–3* are relatively rare in prostate cancer, while gene amplification has been observed in up to 4.5% (*AKT1*), 2% (*AKT2*), and 4.7% (*AKT3*) of prostate cancer cases, respectively [144]. The importance of each AKT isoform in PTEN-deficient prostate cancer remains unclear, with some preclinical data suggesting a higher dependency on AKT2 compared to AKT1 [193], yet only deletion of *Akt1* and not *Akt2* has been shown to suppress prostate neoplasia in a *Pten^+/−^* transgenic mouse [194,195]. Interestingly, overexpression of AKT3 in *PTEN*-null PC-3 mCRPC cells can promote proliferation and tumor growth in vivo [196], whereas systemic deletion of *Akt3* has been shown to trigger neurological conditions, including depressive and anxiety-like behaviors in mice [197]. In contrast, systemic deletion of *Akt1* or *Akt2* in mice increases mortality, due to significant disruptions in circulating glucose and insulin levels, resulting in hyperglycemia, which is notably a known adverse effect of pan-AKT inhibitors in the clinic [198]. However, manageable clinical safety profiles are now being established for pan-AKT inhibitors administered alone and in combination with established taxane-based or androgen/AR-targeted therapies, with patient responses linked to dysregulated PTEN/PI3K signaling in several solid tumors (including prostate cancer) [199,200,201,202,203,204,205]. Preclinical studies have also supported the stratification of PTEN-deficient prostate cancer patients for AKT inhibitor treatment [206,207,208,209,210], yet activation of potential therapeutic resistance pathways has been identified (discussed below, [180,207,211,212]). These findings underscore the need to further explore the molecular mechanisms underlying AKT-inhibitor resistance to aid the discovery and development of novel therapeutic strategies that can improve patient outcome, including combination therapies.

Pan-AKT inhibitors that are currently in clinical testing include capivasertib (AZD5363), MK-2206 and ipatasertib (GDC-0068), with preclinical data showing increased sensitivity in various PTEN-deficient cancer lines and xenograft models (including prostate cancer) [208,209,210]. Moreover, capivasertib monotherapy in PTEN-deficient intact or castrated prostate cancer mouse models has shown strong anti-tumor effects, and improves survival in an aggressive prostate cancer model driven by PTEN/p53 loss [207]. Similarly, MK-2206 was shown to significantly reduce tumor growth in a PTEN-deficient prostate cancer model text, mTOR activity was not suppressed suggesting that mTOR signaling is regulated independently of AKT in this setting [206]. Dose-dependent responses in *PTEN*-null and PTEN-low prostate cancer lines have also been observed following ipatasertib treatment, further supporting the finding that AKT inhibition can inhibit PTEN-deficient prostate cancer growth [210].

Clinical evaluation in mCRPC patients has confirmed that capivasertib is tolerable in combination with docetaxel and enzalutamide, with the latter combination showing clinical responses in patients with PTEN loss [199,200]. Follow-up phase II studies are currently ongoing using these combinations, which could provide further insight into how these therapies could be tailored to benefit patients (NCT04087174 and NCT02525068). Ipatasertib combined with abiraterone is also reported to have a good tolerability profile in mCRPC patients [213], and another active study will test the safety and therapeutic benefit of ipatasertib in combination with docetaxel (NCT04404140). Interestingly, a phase II study combining ipatasertib and abiraterone has reported reduced rPFS in mCRPC patients, with a greater response observed in those with PTEN loss (NCT01485861) [214]. Early data presented at the European Society of Medical Oncology (ESMO) 2020 virtual annual meeting from the ongoing phase III IPATential150 study (NCT03072238) showed similar promising responses in PTEN-deficient prostate cancer using the same treatment combination (with the addition of prednisone to the abiraterone arm). Another ongoing clinical trial is also testing MK-2206 in combination with bicalutamide in high-risk prostate cancer patients post-surgery, radiotherapy or cryoablation (NCT01251861), and two structurally similar AKT inhibitors, uprosertib (GSK2141795) and afuresertib (GSK2110183), are also being tested clinically. To date, uprosertib has shown partial responses in two patients with either a *PIK3CA* mutation or PTEN loss (by IHC) in a phase I study in solid tumors [215], while afuresertib is currently under investigation in mCRPC patients (NCT04060394).

Taken together, the recent clinical data from studies exploring AKT inhibitors in prostate cancer is promising, particularly in combination with androgen/AR blockade. Furthermore, PTEN loss is emerging as a key biomarker for patient response. Nevertheless, despite clinically manageable safety profiles being reported, toxic side effects (including hyperglycemia and diarrhea) associated with PI3K/AKT inhibition remain a concern for patients and clinicians, and new approaches to overcome this issue are in demand. Interestingly, the use of metformin in combination with AKT inhibition has been suggested to decrease insulin levels and limit these adverse side effects [192,216], while similar results have been observed with PI3K inhibitors used alongside a ketogenic diet [217].

### 3.4. mTOR Inhibition

The mTORC1 and mTORC2 signaling pathways play an important role in mediating PI3K/AKT signaling by regulating AKT activity directly (mTORC2) and indirectly (mTORC1) [218]. A number of feedback loops also exist between the mTORC1 and mTORC2 pathways enabling cross-regulation [218]. Structurally, both complexes center around the protein kinase mTOR, mammalian lethal with SEC13 protein 8 (mLST8) and DEP domain-containing protein 6 (DEPTOR). However, they differ in additional components [219]. For example, the mTORC1 complex includes the regulatory-associated protein of mTOR (RAPTOR) and the proline-rich AKT substrate of 40 kD (PRAS40), while mTORC2 contains the rapamycin-insensitive companion of mTOR (RICTOR) and mammalian stress-activated protein kinase-interacting protein 1 (MAPKAP1/mSIN1) [219]. These structural differences allow each complex to perform distinct functions, which ultimately promote AKT signaling and facilitate tumor growth and progression. With the exception of *DEPTOR* gene amplification, genetic alterations to mTORC1/2 components are relatively infrequent in prostate cancer [144]. However, both complexes are commonly activated via upstream activation of the pathway [144]. Paradoxically, amplification of *DEPTOR* has been shown to correlate with worse progression-free survival [144], while a reduction in DEPTOR protein and mRNA transcript levels has also been implicated with prostate cancer progression [220]. Furthermore, systemic deletion of *Deptor* in a *Pten*^+/−^ transgenic mouse was shown to promote prostate tumorigenesis accompanied with increased mTORC1/2 signaling, suggesting that the restoration of *DEPTOR* could be a beneficial therapeutic approach for PTEN-deficient prostate cancer [220].

Deciphering the role of mTORC1 in PTEN-deficient prostate cancer has been largely assessed through a series of studies using sirolimus (rapamycin) and its analogues (rapalogs), which potently inhibit mTORC1, with minimal effect on mTORC2, unless under prolonged exposure [221,222]. Short-term treatment with rapamycin in *Pten*^+/−^ mice on a C57BL/6 background can suppress mTORC1 activity and inhibit the formation of prostate tumorigenesis, suggesting that tumor growth is mTOR dependent in this setting [223], and this anti-tumor effect is further supported by several independent studies in other *Pten*-deficient cancer models treated with rapamycin analogues [224,225,226,227,228]. Conditional co-deletion of *Pten* and tuberous sclerosis 1 (*Tsc1*), which negatively regulates mTORC1 activity, has also been shown to promote tumor growth in a liver cancer mouse model [229]. Further work has also indicated that inactivation of tuberous sclerosis 2 (TSC2) is required for PTEN-dependent prostate tumor growth [230]. However, two separate studies have rebuked this finding by showing that there is no cooperation between *Tsc2/Pten* co-deletion during prostate tumorigenesis in mice [223,231]. Hence, the functional role and therapeutic benefit of targeting TSC1/TSC2 in PTEN-deficient prostate cancer remains unclear [232].

Interestingly, mTORC2, and more specifically RICTOR, is reported to be required for PTEN-deficient prostate tumorigenesis in mice [233], suggesting that targeting RICTOR/mTORC2 may be efficacious against PTEN-deficient prostate cancer in the clinic [233]. Dual mTORC1/2 inhibitors have also been developed that may overcome mTORC1 therapeutic resistance via oncogenic mTORC2 signaling [234,235]. Encouragingly, the dual mTORC1/2 inhibitor AZD8055 (discontinued) can significantly inhibit cell growth in rapamycin-resistant PTEN-deficient prostate cancer cells in vitro [236], and the dual mTORC1/2 inhibitors vistusertib (AZD2014), RapaLink-1 and sapanisertib (INK-128, MLN0128) are reported to significantly decrease tumor growth in PTEN-deficient prostate cancers in patient-derived xenograft and/or transgenic mouse models, further adding to the appeal of targeting mTORC1/2 clinically [237,238,239]. Notably, in patients receiving a tolerable 2 week course of vistusertib treatment prior to radical prostatectomy is reported to correlate with mTORC1/2 inhibition within tumor tissues and PSA reductions in 15% of patients (NCT02064608), although the development of this small-molecule inhibitor has since been discontinued [240]. A clinical trial investigating the dual mTORC1/2 inhibitor sapanisertib in patients with mCRPC was also closed early due to suspected AR activation in response to treatment, and little clinical response was observed (largely due to dose-limiting toxicity and tolerance) (NCT02091531) [39]. Thus, further exploration into the tumor biology and to the mode of action of mTORC1/2 inhibitors is needed to discover efficacious approaches for these small-molecule inhibitors, either as single agents or in combination with current treatment regimens.

So far, much of the clinical focus towards mTOR inhibition has revolved around sirolimus (rapamycin) and its analogues everolimus (RAD001) and temsirolimus (CCI-779). However, despite showing good tolerability profiles when used alone or combined with other treatments, little anti-tumor activity has been reported in the clinic [40,41,42,241,242,243,244,245,246,247,248,249]. Efforts to improve the bioavailability and performance of these agents through nanoparticle encapsulation and binding are ongoing, although whether this improves their anti-tumor effects remains to be determined [250,251]. Nonetheless, early reports from a separate study have indicated promising responses in patients with PTEN-deficient mCRPC (NCT00976755). In this small phase 2 study, PTEN loss was predictive of response in chemotherapy naïve mCRPC patients treated with the mTORC1 inhibitor everolimus daily, with PFS in *PTEN*-deleted patients improved compared to unselected patients [252]. Furthermore, a recent case study involving a patient with a *PTEN* inactivating mutation treated with everolimus showed prolonged disease stabilization after previously failing rounds of bicalutamide, docetaxel, abiraterone, and cabazitaxel treatment [253]. Interestingly, the combination of everolimus alongside bicalutamide has shown some encouraging clinical responses in bicalutamide-naïve CRPC patients, but not in those with prior exposure to the drug [254,255]. Further clinical exploration is planned to test the use of everolimus alongside other androgen/AR-directed therapies such as apalutamide (NCT02106507) and enzalutamide (NCT02125084). However, patients will not be selected via genetic status prior to treatment. It will therefore be interesting to see whether findings from these trials also reveal a correlation between response and PTEN deficiency (or any other PI3K–AKT–mTOR pathway genetic drivers). Interestingly, a clinical trial involving everolimus in patients with CRPC that have a genetic alteration in the PI3K–AKT–mTOR pathway (identified by next-generation sequencing) is ongoing (NCT03580239). This approach will gain further insight into the value of everolimus as a precision medicine to target PTEN-deficient prostate cancer.

### 3.5. Approaches to Enhance the Efficacy of PI3K–AKT–mTOR-Directed Therapy

Various mechanisms of intrinsic and acquired resistance to PI3K–AKT–mTOR-directed therapies have been identified that prevent complete suppression of the pathway, such as feedback loops and redundancy within the cascade, which could be targeted to increase drug sensitivity [211,212]. For instance, increased activity of kinases that act in a redundant fashion to regulate AKT-mediated downstream effector cascades (e.g., mTORC1) can facilitate tumor cell resistance to AKT inhibition [180,206,256,257]. Interestingly, one study has reported that AKT-independent activation of mTORC1 following AKT inhibition can be overcome by combining AKT and mTORC1 inhibitors in a PTEN-deficient prostate cancer transgenic mouse model [206], supporting the concept of vertically targeting the PI3K–AKT–mTOR pathway to prevent drug resistance associated with PI3K–AKT–mTOR-directed monotherapies [180]. Below, we discuss several current approaches being explored to overcome resistance to PI3K–AKT–mTOR-targeted therapies.

#### 3.5.1. SGK Inhibition

SGKs, which belong to the same family of AGC (protein kinase A/protein kinase G/protein kinase C) serine/threonine kinases as AKT, are becoming increasingly recognized as an important PI3K downstream effector family of kinases [258]. The SGK family is comprised of SGK1, SGK2 and SGK3 isoforms, which are activated through PDK1 and mTORC2 phosphorylation in a similar manner to AKT [259,260]. However, SGKs can act independently to AKT to direct AKT-mediated signaling events through the regulation of shared downstream substrates [258]. Consequently, they are also susceptible to hyperactivation following PTEN loss and can contribute to tumor growth [190]. However, the relationship between SGKs and PTEN status in prostate cancer is yet to be fully established. Compensatory activation of AKT downstream targets (including mTORC1 in breast and colorectal cancer cell lines) by SGK1 has been observed in response to AKT blockade, suggesting that SGKs may mediate AKT-driven tumorigenic processes in the presence of an AKT inhibitor [258,261,262,263]. Interestingly, breast cancer cell lines resistant to the AKT inhibitors capivasertib and MK-2206 express high levels of SGK1 and are sensitive to SGK knockdown (KD) [264], while sustained AKT inhibition leads to increased SGK3 expression that can replace AKT-mediated activation of mTORC1 [257]. Furthermore, *SGK1*/*SGK3* are amplified in up to 3% and 20% of prostate cancer cases, respectively [144], and *SGK1* has been identified as an AR-regulated target gene [256]. Significantly, SGK1 inhibition is reported to reduce prostate cancer cell line proliferation and invasive potential, and can synergize with the mTOR inhibitor rapamycin in vitro [256,265]. In vivo preclinical studies have also shown that *SGK1* KD in PC-3 xenografts significantly reduces tumor burden and metastatic potential compared to controls [265], and combining SGK and AKT inhibitors (e.g., 14h and MK-2206) is synergistic in both breast and thyroid cancer models [257,266,267]. These data suggest that dual inhibition of both AKT and SGK could be an effective therapeutic approach to overcome AKT inhibitor resistance. However, SGK inhibitors are yet to advance into clinical trials.

#### 3.5.2. PKC Inhibition

In addition to SGKs, the PKC superfamily of isoenzymes presents another set of PI3K downstream effector proteins that can also be regulated by mTORC2 [268]. Three subfamilies of PKCs exist which are classified as either conventional (α, βΙ, βΙΙ, and γ), novel (δ, ε, η, and θ) or atypical (ζ, μ, and λ/ι) depending on their lipid-activation profiles, although each member can be activated by PIP3 in the presence of PKC co-factors [189,269,270,271]. The role of these isoenzymes in cancer progression remains controversial, with several members shown to mediate both pro-tumorigenic and tumor-suppressive functions (reviewed in [272]). This is highlighted by the atypical PKC member PKCζ that can mediate both pro- and anti-tumorigenic responses in prostate cancer [273,274,275,276]. Genetic inactivation of *PRKCZ* that encodes PKCζ facilitates invasive prostate carcinoma progression in *Pten*^+/−^ mice, indicating that PKCζ is a tumor suppressor in the context of PTEN deficiency [275]. However, contradictory to this, a reduction in tumor growth and metastatic burden has been observed following PKCζ suppression in PC-3 xenografts, attributable in part to impaired AKT signaling [277]. Interestingly, overexpression of the pro-oncogenic kinase and novel PKC member PKCε can also promote prostate epithelial cell growth and invasion in *Pten* heterozygous transgenic mouse models, associated with NF-κB activation and COX-2 upregulation [278,279]. In support, treatment with either the Cox-2 inhibitor rofecoxib or the NF-κB inhibitor parthenolide significantly abrogated tumor growth of a xenograft model using *PTEN*-null prostate epithelial murine cells engineered to overexpress PKCε relative to control mice [279], and long-term rofecoxib administration in a transgenic *Pten*^+/−^ mouse conditionally overexpressing PKCε in the prostate epithelium inhibited tumor formation [279]. These findings build upon previous work showing that shRNA-mediated PKCε KD in a highly metastatic clone of PC-3 cells could inhibit metastatic dissemination to the bone in vivo [280]. In vitro invasion assays have also revealed PKCε silencing or treatment with the PKCε inhibitor εV1-2 impairs invasive potential [280]. Thus, although our molecular understanding of the role of PKCs in prostate cancer is limited, these studies provide key evidence that targeting specific PKC isoenzymes may hold therapeutic promise.

#### 3.5.3. GTPase Inhibition

The Rho family of small GTPases including RAC proteins (RAC1, RAC2 and RAC3) and CDC42 are important mediators of cell cytoskeletal rearrangements that regulate cell migration and metabolism [281,282]. Small GTPase activity is governed by guanine nucleotide exchange factors (GEFs), which convert inactive GDP-bound GTPases into an activated GTP-bound state. This process is tightly controlled by upstream regulatory networks, including AKT-independent PIP3-mediated PI3K signaling [191,283]. Interestingly, both RAC and CDC42 can directly activate p110β by directly binding to its RAS-binding domain (RBD), and RAC1 activity has also been shown to be regulated by p110β in a PTEN-deficient mouse model of hematological cancer [284,285]. We have also previously shown that PTEN-deficient prostate cancer growth is at least partly driven through p110β-RAC1 activation in vivo [21]. Hyperactivation of RAC1 and CDC42 in response to PTEN loss has also been linked to increased cell motility in mouse fibroblasts [286], possibly linked to PTEN’s ability to suppress PIP3-dependent Rac exchanger 2 (PREX2) activity, a GEF that activates RAC1 to promote cell migration and invasion [287]. Thus, the p110β-RAC1 signaling axis may have important therapeutic implications for PTEN-deficient prostate cancer.

Both RAC1-specific and dual RAC1/CDC42 inhibitors (NSC23766 and AZA1, respectively) have shown preclinical efficacy in prostate cancer cell lines (including *PTEN*-null PC-3 cells), inhibiting both cell migration and in vivo tumor growth [288,289]. In support, siRNA silencing of RAC/CDC42 regulating GEFs, such as PIP3-dependent Rac exchanger 1 (PREX1) and vav guanine nucleotide exchange factor 3 (VAV3), can significantly reduce prostate cancer cell proliferation and migration in vitro [290,291]. Furthermore, exogenous expression of PREX1 in CWR22Rv1 human mCRPC cells that have limited metastatic potential in vivo caused spontaneous lymph node metastasis in mice, with no effect on primary tumor growth [290]. Thus, inhibition of GTPase–GEF interactions presents an attractive avenue for therapeutic intervention, particularly as an anti-metastatic approach. The small-molecule inhibitor ZCL278 that blocks CDC42–intersectin (ITSN) interactions and its more potent analogue ZCL367 that also inhibits RAC1 have shown preclinical promise in prostate cancer [292,293]. This includes their ability to inhibit cell proliferation and migration in a variety of prostate cancer cell lines including PC-3 (*PTEN^−/−^*) and DU145 (*PTEN^+/−^*) cells in vitro, and ZCL367 is also reported to significantly inhibit tumor growth in a xenograft model of lung cancer [292]. Intriguingly, both RAC1 activity and CDC42 activity have been associated with therapeutic resistance in different cancer types, including enzalutamide resistance in CRPC, and RAC/CDC42 inhibition has been suggested as a potential approach to overcome resistance to PI3K–AKT–mTOR-targeted treatments [294,295,296,297,298]. Although RAC/CDC42-specific inhibitors remain to be explored clinically, one FDA-approved non-steroidal anti-inflammatory drug (NSAID), R-ketorolac, has been shown to potently inhibit RAC1 and CDC42, and has been demonstrated to improve ovarian cancer patient survival (NCT02470299), suggesting that GTPase targeting could be efficacious in other human cancers with elevated expression and activity of RAC1 and/or CDC42 [299].

#### 3.5.4. AXL Inhibition

The RTK AXL (tyrosine-protein kinase receptor UFO) has been heavily implicated in various aspects of tumorigenesis in several cancer types, and is overexpressed in prostate cancer [300,301]. AXL makes up part of the TAM family of receptors that also includes TYRO3 protein tyrosine kinase (TYRO3) and MER proto-oncogene tyrosine kinase (MER), which promote cell proliferation, migration and survival in response to upstream activation via extracellular growth factors, such as growth arrest-specific protein 6 (GAS6) [302]. In prostate cancer, GAS6-AXL signaling has been shown to promote proliferation by inducing AKT and mitogen-activated protein kinase (MAPK) phosphorylation [303]. In addition, GAS6-mediated activation of AXL has been shown to promote EMT and cell migration in DU145 cells, and can protect PC-3 cells from undergoing chemotherapy-induced apoptosis [304,305]. AXL activation has also been shown to initiate a dormant state in prostate cancer cells. However, the importance of AXL in maintaining long-term dormancy and its role during metastasis have been debated in recent follow-up work [305,306].

AXL signaling has also been implicated in promoting resistance to PI3K–AKT–mTOR pathway-directed therapy in various settings. For instance, AXL-mediated activation of mTORC1 signaling through PKC has been shown to promote acquired resistance to the p110α inhibitor BYL719 in squamous carcinoma cell lines in vitro and in vivo, while AXL activation has also been observed in an oesophageal cancer cell line resistant to AKT inhibition [307,308]. Interestingly, AXL is upregulated and activated in docetaxel-resistant prostate cancer cell lines, and AXL blockade in these cells, either by siRNA targeting or treatment with the non-specific tyrosine kinase inhibitor amuvatinib (MP470), induced apoptosis and reduced cell migration and invasion [309]. Amuvatinib treatment also suppressed tumor growth in a docetaxel-resistant prostate cancer xenograft model, and significantly enhanced the efficacy of docetaxel [309]. This inhibitor has been tested clinically in advanced solid cancers in combination with different chemotherapeutics and appears to be well tolerated, with anti-tumor benefits reported, particularly in lung neuroendocrine tumors, and stable disease was observed in three of the four prostate cancer patients recruited (NCT00881166) [310]. Combining amuvatinib and the epidermal growth factor receptor (EGFR) inhibitor erlotinib has also been shown to significantly inhibit tumor growth in *PTEN*-null LNCaP xenografts, accompanied by reduced AKT phosphorylation at S473 and T308 [311]. Several more specific inhibitors have also been developed that target both the AXL receptor directly and its ligand GAS6, including bemcentinib (BGB324) [312]. This inhibitor was also shown to induce apoptosis in docetaxel-resistant prostate cancer cell lines in vitro, at least partly through reducing levels of phosphorylated AKT, and was also able to partially rescue an EMT phenotype driven through docetaxel resistance [309]. In addition to the pro-tumorigenic role of AXL on tumor cells, AXL signaling can also suppress anti-tumor immune responses. Therefore, these inhibitors may advantageously play a dual role both directly killing cancer cells and improving immune cell-mediated killing [313].

Clinical trials are now testing bemcentinib in several cancers and early data suggest that it is well tolerated. However, to date, no trials have investigated its efficacy in prostate cancer [312,314]. Although little work has explored the role of AXL inhibition within the context of PTEN status, one study has shown that selective cytotoxic responses are achieved in PTEN-deficient glioblastoma cells when S6K1 inhibitor LY-2779964 is combined with AXL/TAM kinase inhibitor BMS-777607 or TAM kinase KD, suggesting that the combined targeting of S6K1 and TAMs could be a potential strategy for treatment of PTEN-deficient tumors [315]. In contrast, AXL inhibition in *BRAF* mutant melanoma may be more effective in PTEN wild-type patients, as phosphorylated AXL is only significantly increased in BRAF inhibitor-resistant cell lines in the context of endogenous PTEN [316]. Therefore, while preclinical data indicate that AXL is a promising therapeutic target, particularly within docetaxel-resistant disease, more research is required to establish the role of AXL activation in prostate cancer, and to determine whether PTEN status is a predictive marker for AXL activity. Given the clear association between AXL signaling and drug resistance, including PI3K–AKT–mTOR pathway inhibitors, future work exploring AXL inhibitors could also provide new insights into how therapeutic resistance can be overcome.

## 4. PTEN and the DNA Damage Response

DNA damage events are common and may result from both endogenous processes, such as errors occurring during DNA replication and metabolic stress, and exogenous sources, such as ionizing radiation, drugs and toxins [317]. In order to maintain genomic integrity and prevent tumorigenesis, cells have developed a range of complex mechanisms to detect DNA damage and initiate a series of downstream effector cascades that, together with the action of cell cycle checkpoints, culminate in either cell cycle arrest, targeted repair/bypass of the damage or, if the former are not possible, cell death or senescence [318]. Collectively, these mechanisms are known as the DNA damage response (DDR), of which five main pathways have been described. Base excision repair (BER) corrects small base lesions such as modified bases at basic sites and DNA single-strand breaks (SSBs) that arise as a consequence of deamination, oxidation or alkylation caused by chemicals, ionizing radiation and spontaneous DNA decay [319]. Nucleotide excision repair (NER) acts to remove a number of structurally unrelated DNA lesions, such as 6-4 pyrimidine-pyrimidone photoproducts (induced by UV radiation), bulky chemical adducts and intrastrand crosslinks. Mismatch repair (MMR) corrects DNA base–base mismatches that arise during normal DNA metabolism or aberrant DNA processing during replication, recombination and repair [320]. Homologous recombination repair (HRR) is one of two major repair mechanisms for DNA double-strand breaks (DSBs), highly toxic lesions that are induced by replication errors, ionizing radiation and chemicals [321]. The process of HRR comprises a series of interconnected pathways that, through the use of the sister chromatid as a template, culminate in the error-free restoration of the original DNA code [322]. Non-homologous end joining (NHEJ) is the other major repair mechanism for DSBs. Unlike HRR, NHEJ mechanisms do not utilize a homologous template and therefore occur during all phases of the cell cycle, rather than being restricted to S and G2 phases alone [323]. The lack of template renders classical NHEJ error prone, which in turn can lead to DNA rearrangements that may drive genomic instability and oncogenic transformation [324].

Whilst originally considered to be a cytoplasmic protein, PTEN is now known to also localize to the nucleus and associate with centromeres, where it can exert several functions, including maintenance of chromosomal integrity and regulation of the DDR [325]. Previous work has shown that bi-allelic deletion of *Pten* in mouse embryonic fibroblasts (MEFs) induces spontaneous DNA DSBs, thereby indicating that PTEN plays a role during DSB repair and genomic stability [17]. In this study, transcription of *RAD51* (that encodes a protein vital to DSB repair via HRR) was significantly reduced in *Pten*-null MEFs while enforced expression of RAD51 restored DSB repair, suggesting that PTEN regulates DSB repair through transcriptional regulation of *RAD51* [17]. In support, ectopic expression of PTEN in human *PTEN*-null PC-3 CRPC cells induced *RAD51* transcription and RAD51 protein expression, and reduced the number of DSBs [17]. However, although an independent study also revealed that PTEN depletion in DU145 mCRPC cells causes increased DNA DSBs and reduces HRR following irradiation, no significant correlation between RAD51 protein level and PTEN status was detected in a large radical prostatectomy tissue microarray (TMA) (n > 1500) [326]. Similar non-correlative findings have also been reported in a separate primary prostate cancer TMA, and further work has shown that RAD51 recruitment to DSBs in prostate cancer cells in response to UV irradiation is not impeded by PTEN deficiency [327]. These conflicting findings indicate that whilst it is clear PTEN plays a role in the repair of DSBs via HRR, further research is needed to elucidate the exact molecular mechanisms and pathways involved.

PTEN has also been implicated in cell cycle regulation through interactions with cyclin D1 and checkpoint kinase 1 (CHK1, or CHEK1), both of which play crucial roles in cell cycle checkpoint activation to facilitate DNA damage recognition and repair. For example, siRNA-mediated KD of *PTEN* in DU145 cells has been shown to diminish CHK1 activation in response to UV and X-ray irradiation, with a resulting reduction in cell cycle arrest at the G2/M checkpoint [326]. Interestingly, this effect appears to be mediated via AKT, which can phosphorylate CHK1 at S280, resulting in reduced CHK1 nuclear localization and hence impaired checkpoint activation that can be rescued following AKT inhibition [326]. As AKT-dependent phosphorylation of CHK1 is increased in PTEN-depleted cells, these findings indicate that PTEN exerts its cell cycle activity through the regulation of AKT activity, and the subsequent phosphorylation and CHK1 cytoplasmic sequestration [326,328]. In addition to DSB repair and cell cycle regulation, PTEN is also reported to be essential for the efficient repair of UVB related DNA damage products such as pyrimidine(6-4)pyrimidone dimers via the process of NER [329], suggesting that PTEN is involved in multiple arms of the DDR. This adds further complexity to the range of PTEN nuclear tumor-suppressive functions and may in turn indicate a role for DDR-targeting treatment strategies in patients with aberrations in PTEN.

In preclinical pharmacological studies, PTEN loss has been shown to increase sensitivity to DDR-targeting agents such as poly-ADP ribose polymerase (PARP) inhibitors in a range of cancer cell lines, and in the *PTEN*-null colorectal cancer HCT116 xenograft model [330]. However, studies in the setting of PTEN-deficient prostate cancer have been conflicting. Whilst an early report did not demonstrate a relationship between decreased PTEN expression and sensitivity to PARP inhibition in metastatic prostate cancer cell lines in vitro [327], a more recent study reported that PTEN KD in DU145 cells increases sensitivity to the PARP inhibitor olaparib (AZD2281) when compared with control cells [326]. Furthermore, whilst two early phase clinical trials involving patients with heavily pretreated CRPC have not demonstrated a correlation between PTEN loss and response to PARP inhibition [331,332], patient numbers were low and therefore larger-scale studies are required to further delineate the relationship between PTEN status and PARP inhibitor sensitivity in the clinic.

Whilst the jury remains out on whether PTEN loss confers increased sensitivity to PARP inhibitors, it is generally considered that PARP inhibition results in increased AKT pathway activation as a form of cytoprotective response in a variety of disease model systems [333,334,335,336]. Additionally, as discussed above, AKT-dependent phosphorylation and cytoplasmic sequestration of CHK1 appears to be enhanced in PTEN-deficient cells, indicating that PTEN controls the G2/M cell cycle checkpoint through its regulation of AKT activity [326,328]. Taken together, these findings indicate that combination therapy with a PI3K–AKT–mTOR pathway inhibitor in patients with advanced prostate cancer may overcome the cytoprotective effects of PARP inhibitor-induced AKT activation, perhaps with additional benefit in those with PTEN deficiency and increased AKT signaling. Whilst preclinical studies and early phase clinical trials have demonstrated promise for combined PARP and PI3K–AKT–mTOR pathway blockade in patients with both HRR-proficient and -deficient cancers (determined by functional status of breast cancer susceptibility protein 1 and 2, BRCA1 and BRCA2) [337,338,339,340,341], it has not yet been explored in prostate cancer specifically. However, a phase I/II trial evaluating the safety and efficacy of the AKT inhibitor ipatasertib in combination with the PARP inhibitor rucaparib in patients with advanced breast, ovarian and prostate cancer is currently recruiting and should provide invaluable insight into the efficacy of this combination in the setting of advanced prostate cancer (NCT03840200).

## 5. PTEN and the Tumor Microenvironment

Studies addressing the complex relationship between tumor cells and components of the TME (e.g., various cancer-associated fibroblast populations, smooth muscle cells, structural matrix factors, vasculature, nerve cells and the immune cell infiltrate) have revealed that the TME can influence tumor cell behavior at every stage of tumorigenesis, from tumor initiation through to metastatic progression [342,343,344]. The generation of new and improved models to study the TME has informed and facilitated the development of new therapeutics that target different TME components, and aided the discovery of predictive biomarkers within the TME that may be used to guide personalized treatment decisions [345]. PTEN represents one such biomarker that has been shown to influence the behavior of several components of the TME in different cancer types including prostate cancers, discussed below.

### 5.1. PTEN and Tumor–Stroma Interactions

Communication between the prostate epithelial cells and the surrounding stroma is crucial for prostate organogenesis and the maintenance of normal adult tissue homeostasis, and has been shown to play a key role during ageing and prostate tumorigenesis [343]. Although the majority of anti-cancer therapeutics specifically target the tumor cells, TME components can promote tumor cell resistance to these agents, as tumor–stroma interactions (TSIs) have been shown to influence both tumor growth rates and treatment responses, including in the context of radiotherapy, surgery, chemotherapy and immunotherapy [344,346,347,348,349,350,351]. For instance, the physical interaction of breast cancer cells and mesenchymal stem cells can confer resistance to trastuzumab in vitro by activation of non-receptor tyrosine kinase c-SRC and PTEN downregulation [351], and analysis of a prostate cancer TMA has revealed that stroma volume and altered expression of desmin and smooth muscle α-actin are each independent and significant predictors of biochemical recurrence [352]. In addition, resistance to EGFR inhibition is reported to be caused by hepatocyte growth factor (HGF) secretion by fibroblasts in triple-negative breast cancer, which activates EGFR-MET crosstalk [353], while the co-culture of prostate cancer cell lines with cancer-associated fibroblasts (CAFs) can mediate resistance to the multityrosine kinase inhibitor sorafenib [354]. Although the functional consequence of PTEN loss in prostate epithelial cells and its role during prostate cancer progression have been well characterized, the tumor-suppressive role of PTEN in the prostate TME remains unclear. Intriguingly, increased extracellular matrix stiffness has been shown to induce miR-18a expression to reduce PTEN levels by downregulating homeobox A9 (HOXA9) in human and mouse breast cancer tissue, and matrix stiffness and high miR-18a expression can predict for poor prognosis in patients with luminal breast cancers [355]. Whether a similar association occurs in prostate cancer remains to be determined.

PTEN loss in tumor cells can also result in remodeling of the stromal compartment in prostate cancer [356]. Conditional *Pten* homozygous deletion in the prostate epithelium of castrated mice is reported to loosen areas of surrounding smooth muscle actin and promote collagen deposition, increasing invasive potential [356]. Interestingly, deletion of *Pten* in stromal fibroblasts within the murine mammary gland has been shown to initiate significant extracellular matrix (ECM) remodeling, resulting in mammary tumor initiation and invasive progression through alterations in collagen alignments [357,358]. These findings indicate that PTEN can elicit its tumor-suppressive function through stromal fibroblasts and raise the possibility of a similar role in other epithelial cancers. Although stromal-specific deletion of *Pten* has not been assessed in the prostate, *p62* deletion in this compartment leads to metabolic reprogramming of prostate stromal fibroblasts, inactivating the p62/mTORC1/c-Myc signaling network that promotes inflammation and tumorigenesis [359]. Ultimately, these findings illustrate the need for further research to investigate how PTEN loss promotes pro-tumorigenic prostate TSIs, ECM remodeling and an immunosuppressive microenvironment during prostate tumorigenesis and in response to therapeutic intervention.

### 5.2. PTEN and the Immune Response

Loss of the tumor suppressor PTEN in both epithelial or surrounding stromal/immune cells can also have an overall immunosuppressive effect on the TME via multiple direct and indirect mechanisms [360,361]. For example, intrinsic PTEN loss in established melanoma cell lines and patient-derived cells has been shown to promote PI3K-mediated activation of immunosuppressive cytokines [362]. Similarly, in silico screening of infiltrating immune cells from 741 primary and 96 metastatic clinical prostate cancer samples linked PTEN deficiency with an immunosuppressed TME [363]. Furthermore, activation of the JAK2/STAT3 pathway resulting in an immunosuppressive TME has been reported in senescent PTEN-null prostate tumors, which supports tumor growth and chemoresistance [364]. Although not well characterized in prostate cancer specifically, direct PTEN loss in several immune cell types, including regulatory T cells, natural killer cells and macrophages, has also been demonstrated to shift the TME to an immunosuppressive state in various cancer models (reviewed in [360,361]).

The enhanced immunosuppressive TME associated with PTEN-deficient prostate tumors is likely to have important clinical implications, especially in the context of immunotherapy. Several immunotherapies are already being used in the clinic to treat patients with metastatic prostate cancer, including the immune boosting vaccine sipuleucel-T and the PD-1 checkpoint inhibitor pembrolizumab [365]. Both approaches have demonstrated therapeutic efficacy in a subset of patients. However, further research is needed to identify which patients are most likely to benefit [366,367]. Although the implications of PTEN loss and response to these treatments in the context of prostate cancer is yet to be evaluated, preclinical and clinical data from other cancer types are beginning to reveal that PTEN loss may desensitize patients to immunotherapy [360,368,369,370,371]. For example, PTEN-deficient melanoma is associated with a poor response to PD-1 inhibitors pembrolizumab and nivolumab that is reported to reflect reduced T-cell tumor infiltration and activity [369]. Interestingly, combining an anti-mouse PD-1 antibody and the p110β isoform-specific inhibitor GSK2636771 significantly reduced tumor growth and survival relative to monotherapy in *Braf*-mutant, *Pten*-null melanoma in vivo, and was associated with increased T-cell infiltration [369]. A similar response has been observed in the same model when combining GSK2636771 and an anti-OX40 targeting antibody [372]. This preclinical work has led to an ongoing clinical trial (NCT03131908) assessing the safety and efficacy of checkpoint inhibition combined with GSK2636771 in PTEN-deficient metastatic melanoma. Current reports, however, indicate that a phase II arm of this study assessing the treatment of other solid cancers (including prostate cancer) has been discontinued, possibly owing to early data revealing dose-limiting renal toxicities [373]. Despite this, efficacy data from this study may still inform the design of future clinical trials investigating a similar treatment combination in PTEN-deficient cancer.

### 5.3. PTEN and Inflammation

Intra-prostatic inflammation is another microenvironmental factor that has been associated with prostate cancer initiation and progression [374,375,376,377]. Exposure of normal prostate epithelium to various internal and external stresses can trigger an initial inflammatory response which ultimately results in a self-propagating cycle that leads to chronic inflammation (reviewed in [377]). This is maintained by various cell types in the TME including myeloid-derived suppressor cells (MDSCs), TAMs and the prostate epithelial cells themselves [378,379,380,381,382]. Interestingly, deletion of *Pten* in murine prostate epithelial cells has been shown to upregulate inflammatory-associated gene expression resulting in the immediate expansion of intraprostatic MDSCs and sustained immune suppression [383]. Furthermore, siRNA and shRNA suppression of *PTEN* in DU145 and 22Rv1 prostate cancer cell lines has also been shown to upregulate *CXCL8* mRNA that encodes the proinflammatory chemokine interleukin 8 (IL8) [384]. This response was enhanced in hypoxic conditions and was also replicated in prostate tissue from mice with heterozygous *Pten* deletion [384]. Co-culture experiments in vitro have also shown that upregulation of CXCL8 following PTEN-loss can potentiate additional chemokine expression in an autocrine and paracrine manner on surrounding stromal cells to further promote prostate tumor progression and invasion [385]. Importantly, the relationship between PTEN loss and CXCL8 upregulation has been validated in clinical prostate samples and could potentially be exploited therapeutically by targeting cellular inhibitor of apoptosis protein-1 (cIAP-1). cIAP1 is an anti-apoptotic protein that is upregulated in prostate cancer cells following CXCL8-mediated TAM infiltration, and cIAP1 inhibition has been shown to be effective in PTEN-depleted prostate cancer cells in vitro [84].

Upregulation of CXCL8 is also a hallmark of the senescence secretory response, known as the senescence-associated secretory phenotype (SASP), which follows senescence induction and has been shown to promote therapeutic resistance in men with prostate cancer [386]. Of note, PTEN loss in prostate cancer cells is also known to induce a p53-dependent senescent phenotype [387,388]. Significant evidence in the literature has identified that DNA-damaging agents promote senescence in both cancer cells, and surrounding non-malignant cells in the TME [389], while senescent CAFs in prostate cancer have been shown to secrete SASP-related factors to promote tumor cell survival, growth and migration post-chemotherapy and radiotherapy [390,391]. Interestingly, mTOR has been shown to regulate this secretory response [392] and consequently, mTOR inhibition in combination with chemotherapy or radiotherapy may suppresses this secretory program and its ability to promote tumor growth. *PTEN*-deficient induced senescence in prostate tumors can also promote the growth of adjacent non-senescent tumor cells, and cause chemoresistance through a SASP-associated mechanism [364]. The development of selective agents that eliminate senescent cells (senolytics) and SASP-modulating agents (senostatics) could therefore potentiate the anti-tumor effects of senescence driven by PTEN-loss in fibroblasts by reducing the pro-tumorigenic effects associated with the upregulation of SASP [393].

Finally, accelerated prostate cancer progression has also been associated with inflammation caused by a high-fat diet in PTEN-deficient prostate cancer mouse models, suggesting that alterations to patient diet and/or exercise regimens may be especially beneficial for those with PTEN-deficient disease [394,395,396]. Although the clinical data available are still not clear on which nutrients may offer the most protection, switching to a ketogenic diet which encourages the body to burn fat may prove to be a beneficial approach [217]. Additional work in mouse models has also demonstrated that this diet or pharmacological suppression of blood glucose levels can improve sensitivity to PI3K pathway inhibition through the inhibition of PI3K-mediated insulin feedback, which could be an interesting approach for PTEN-deficient prostate cancer [217]. In summary, while current evidence in the literature has clearly identified that PTEN plays a tumor-suppressive role in epithelial and stromal cells, additional work is needed to better understand the molecular mechanisms underlying PTEN regulation of the TME and to discover how this can be effectively exploited to treat PTEN-deficient cancers.

## 6. Conclusions

Loss of the tumor suppressor PTEN is a frequent event in prostate cancer that contributes to tumor growth and therapeutic resistance through oncogenic PI3K--AKT--mTOR signaling, increased~genomic instability, impaired DNA damage repair, pro-tumorigenic reprograming of the tumor microenvironment and immunosuppression. Substantial research efforts addressing the underlying molecular biology of prostate cancer continue to define the tumor-suppressive function of PTEN and have resulted in major advances in targeting PTEN-deficient prostate cancer. Promising treatment options include both direct and indirect approaches to restore PTEN function, and~PI3K--AKT--mTOR blockade in combination with either chemotherapy, androgen/AR-directed agents, DDR inhibitors, immuno-oncology drugs or additional PI3K--AKT--mTOR-directed therapies that may enhance therapeutic efficacy through diverse synergistic and complementary mechanisms. New PI3K--AKT--mTOR inhibitors with increased potency and selectivity offer further promise, along with next-generation delivery systems that are under investigation as precision anti-cancer modalities. However, the clinical evaluation of these approaches is needed to determine the most effective, well-tolerated treatment strategy for PTEN-deficient prostate cancer. Recent findings indicate that PTEN status (and perhaps other genetic drivers of the PI3K--AKT--mTOR pathway) presents an invaluable opportunity to guide treatment decisions to improve the clinical management of this patient population, potentially during both the early and advanced stages of this disease. A key question that remains unanswered is whether patients with PTEN-deficient low-risk localized prostate cancer would benefit from radiotherapy and/or surgery. To address this, future clinical investigations that include establishing PTEN status at diagnosis are needed. Nevertheless, exploiting the predictive value of PTEN to improve patient care will rely on accurate, stringent testing for PTEN activity, and consideration of the disease subtype, the mechanism by which PTEN has been lost, the co-occurrence of other oncogenic PI3K--AKT--mTOR alterations with PTEN loss, and the extent of tumor heterogeneity is paramount. While advances in artificial intelligence-based algorithms, digital spatial profiling, and the generation of new clinically relevant models of prostate cancer that span a range of different disease subtypes are beginning to address these points, the continued exploration into PTEN function, PI3K--AKT--mTOR inhibition and prostate cancer biology is essential for the identification of new therapeutic approaches and valuable predictive biomarkers that will help to refine future personalized therapies for PTEN-deficient prostate~cancer.

## Abbreviations

ADT	Androgen-deprivation therapy
4EBP1	4E (eIF4E)-binding protein 1
AMO	Antisense anti-miR oligonucleotide
AR	Androgen receptor
BER	Base excision repair
BRCA1	Breast cancer susceptibility protein 1
BRCA2	Breast cancer susceptibility protein 2
C2D	C2 domain
Cas9	CRISPR-associated protein 9,
CHK1	Cyclin D1 and checkpoint kinase 1
cIAP-1	Cellular inhibitor of apoptosis protein-1
CRISPR	Clustered regularly interspaced short palindromic repeat
CRPC	Castrate-resistant prostate cancer
CTC	Circulating tumor cell
CXCL8	C-X-C motif chemokine ligand 8, interleukin 8
DDR	DNA damage response
DEPTOR	Disheveled, EGL-10 and pleckstrin (DEP) domain-containing mTOR-interacting protein
DHT	Dihydrotestosterone
DRE	Digital rectal examination
DSBs	Double-strand breaks
EGFR	Epidermal growth factor receptor
EMT	Epithelial-to-mesenchymal transition
ECM	Extracellular matrix
FISH	Fluorescence in situ hybridization
FOXO	Forkhead box protein O
GAS6	Growth arrest-specific protein 6
GSK3β	Glycogen synthase kinase 3 beta
GPCR	G-protein coupled receptor
HER2	Human epidermal growth factor receptor 2
HRR	Homologous recombination repair
IHC	Immunohistochemistry
IL6	Interleukin 6
IL8	Interleukin 8
ITSN	Intersectin
LNA	Locked nucleic acid
MAN2C1	α-mannosidase 2C1
MAPK	Mitogen-activated protein kinase
MAPKAP1	Mammalian stress-activated protein kinase-interacting protein 1
mCRPC	Metastatic castrate-resistant prostate cancer
MDSC	Myeloid-derived suppressor cells
MEF	Mouse embryonic fibroblast
miRNA	Micro ribonucleic acid
mHSPC	Metastatic hormone-sensitive prostate cancer
mLST8	Mammalian lethal with SEC13 protein 8
MMR	Mismatch repair
mpMRI	Multiparametric MRI
mTOR	Mammalian target of rapamycin
mTORC1	Mammalian target of rapamycin complex 1
mTORC2	Mammalian target of rapamycin complex 2
NER	Nucleotide excision repair
NF-κB	Nuclear factor kappa light chain enhancer of activated B cells
NHEJ	Non-homologous end joining
NSCLC	Non-small-cell lung carcinoma
OncomiRs	Oncogenic miRNAs
P	Phosphorylation event
PBD	PIP2-binding domain
PARP	Poly-ADP ribose polymerase
PDK1	Phosphoinositide-dependent kinase 1
PH domain	Pleckstrin homology domain
Phos D	Phosphatase domain
PI3K	Phosphoinositide 3-kinase
PIP2	Phosphatidylinositol 4,5-bisphosphate
PIP3	Phosphatidylinositol 3,4,5-trisphosphate
PKB	Protein kinase B, AKT
PKC	Protein kinase C
PKCε	Protein kinase C epsilon
PKCζ	Protein kinase C zeta
PRAS40	Proline-rich AKT substrate of 40 kDa
PREX1	PIP3-dependent Rac exchanger 1
PREX2	PIP3-dependent Rac exchanger 2
PSA	Prostate-specific antigen
PTEN	Phosphatase and tensin homologue deleted on chromosome 10
RAPTOR	Regulatory-associated protein of mTOR
RBD	Ras-binding domain
RHEB	Ras homolog enriched in brain
RICTOR	Rapamycin-insensitive companion of mTOR
rPFS	Radiographic progression-free survival
RFS	Recurrence-free survival
RTK	Tyrosine kinase receptor
S6K	Ribosomal protein S6 kinase/p70 ribosomal S6 kinase
SGK1	Serum- and glucocorticoid-regulated kinase 1
SGK2	Serum- and glucocorticoid-regulated kinase 2
SGK3	Serum- and glucocorticoid-regulated kinase 3
siRNA	Small interfering RNA
SMIR	Small-molecule inhibitors of miRNA
SRC-3	Steroid receptor co-activator 3
SSBs	Single-strand breaks
TAM	Tumor-associated macrophage
TSC1	Tuberous sclerosis complex 1
TSC2	Tuberous sclerosis complex 2
TSIs	Tumor–stroma interactions
VAV3	Vav guanine nucleotide exchange factor 3
